# Development of minimal physiologically-based pharmacokinetic-pharmacodynamic models for characterizing cellular kinetics of CAR T cells following local deliveries in mice

**DOI:** 10.1007/s10928-022-09818-8

**Published:** 2022-07-22

**Authors:** Chia-Hung Tsai, Aman P. Singh, Cindy Q. Xia, Haiqing Wang

**Affiliations:** 1 Takeda Pharmaceutical Company Limited, 35 Landsdowne St, Cambridge, MA 02139 USA; 2 Renegade Therapeutics Inc, One Broadway 14th floor, Cambridge, MA 02142 USA

**Keywords:** Cellular kinetics modeling, Local delivery, CAR T cell therapy

## Abstract

Chimeric antigen receptor (CAR) T cell therapies have revolutionized the treatment of hematologic malignancies and have potentials for solid tumor treatment. To overcome limited CAR T cell infiltration to solid tumors, local delivery of CAR T cells is a practical strategy that has shown promising therapeutic outcome and safety profile in the clinic. It is of great interest to understand the impact of dosing routes on CAR T cell distribution, subsequent proliferation and tumor killing in a quantitative manner to identify key factors that contribute to CAR T efficacy and safety. In this study, we established mouse minimal physiologically-based pharmacokinetic (mPBPK) models combined with pharmacodynamic (PD) components to delineate CAR T cell distribution, proliferation, tumor growth, and tumor cell killing in the cases of pleural and liver tumors. The pleural tumor model reasonably captured published CAR T cellular kinetic and tumor growth profiles in mice. The mPBPK-PD simulation of a liver tumor mouse model showed a substantial increase in initial tumor infiltration and earlier CAR T cell proliferation with local hepatic artery delivery compared to portal vein and intravenous (i.v.) injections whereas portal vein injection showed little difference from i.v. administration, suggesting the importance of having the injection site close to tumor for maximal effect of non-systemic administration. Blood flow rate in the liver tumor was found to be a sensitive parameter for cellular kinetics and efficacy, indicating a potential role of tumor vascularization in the efficacy of CAR T cell therapies.

**Supplementary Information:**

The online version contains supplementary material available at 10.1007/s10928-022-09818-8.

## Introduction

Chimeric antigen receptor (CAR) T cells are T cells genetically engineered to express an antigen-specific receptor that redirects T cells to tumor-associated antigen (TAA) for subsequent tumor elimination. CAR T cell therapies have demonstrated remarkable efficacy for hematologic malignancies, as shown by the 5 FDA approvals in the last 4 years of autologous anti-CD19 and anti-B cell maturation antigen (BCMA) CAR T cell therapies [1]. The breakthrough of CAR T therapy in hematologic malignancies have prompted numerous efforts in exploring CAR T cell treatment for solid tumors, resulting in drastic increase in clinical trials with CAR T cell therapies. However, CAR T cell treatment of solid tumor has so far not been satisfactory, largely due to limited CAR T cell infiltration into the tumor, which hinders CAR T activation and proliferation, particularly, in the immunosuppressive tumor microenvironment (TME). Novel engineering approaches have been proposed, such as next generation CAR T cell with additional co-stimulatory domain or with armored protein expression [2]. One practical approach to increase CAR T distribution to solid tumor is through local deliveries including hepatic artery infusion, intraperitoneal, intrapleural, intra cavitary, and intratumoral injections. A number of CAR T cell programs, summarized in Table S1, have explored such approach and demonstrated significantly improved efficacy compared to intravenous (i.v.) delivery in mouse tumor models and limited yet promising efficacy and safety profiles in patients. It is, therefore, of great interest to understand the cellular kinetics of CAR T following i.v. and local delivery, and the critical CAR T levels in the tumor that tip the balance of immunity in the tumor microenvironment to trigger CAR T expansion and tumor eradication.

Emerging clinical data of CAR T cells have shown strong correlation between CAR T cell expansion and anti-tumor efficacy [3, 4], where responders are associated with higher maximum CAR T cell concentration (Cmax) and area under the curve (AUC) in blood compared to non-responders. The clinical observation has led to the exploration of such relationship under preclinical settings to facilitate translational effort for future CAR T cell development. To establish the quantitative cellular kinetics-pharmacodynamics (PD) relationship of CAR T cell therapies, mathematical modeling is emerging as a useful tool for cellular kinetics and PD data integration and translation. Earlier models adopted a physiologically-based pharmacokinetic (PBPK) modeling approach and focused on the distribution of non-genetically modified cells of different types of immune cells across species [5]. A recent PBPK model was developed using an elaborative mouse dataset and introduced the concept of retention factor (R) to capture longer residence time in the kidney, liver, and spleen [6]. Modeling effort specifically for CAR T cells has emerged recently as more clinical data became available. Two human cellular kinetics models for anti-CD19 CAR T cells helped determine the characteristic expansion doubling time, the half-lives (t_1/2_) of the contraction and persistence phase, as well as the correlation between the cellular kinetics and cytokine release [3, 7]. On the preclinical side, a mechanism-based PBPK-PD model was developed recently using mouse data for a number of CAR T cells [8]. The model studied the cellular kinetics-PD relationship with much granularity and identified a potential steep dose–response relationship for CAR T cell therapy and a critical role of initial tumor burden in the Cmax of blood cellular kinetics.

The reported PBPK-PD CAR T models have paved the way to explore the difference of CAR T level in solid tumor following i.v. and local administration quantitatively, and the impact on anti-tumor efficacy. In this work, we established mouse minimal PBPK-PD (mPBPK-PD) models focusing on solid tumor-associated organs where CAR T distribution and subsequent expansion are impacted by local blood flow following different administration routes. The minimal PBPK models render flexibility by focusing on organs of interest, and enable addition of PD and efficacy modules to further evaluate the cellular kinetics and efficacy relationship. Our effort helped identify tumor local blood flow rate as one of the factors that determine the efficacy of CAR T cell therapies and provided a conceptual framework that supports local CAR T delivery for solid tumors.

## Methods

**Fig. 1:**
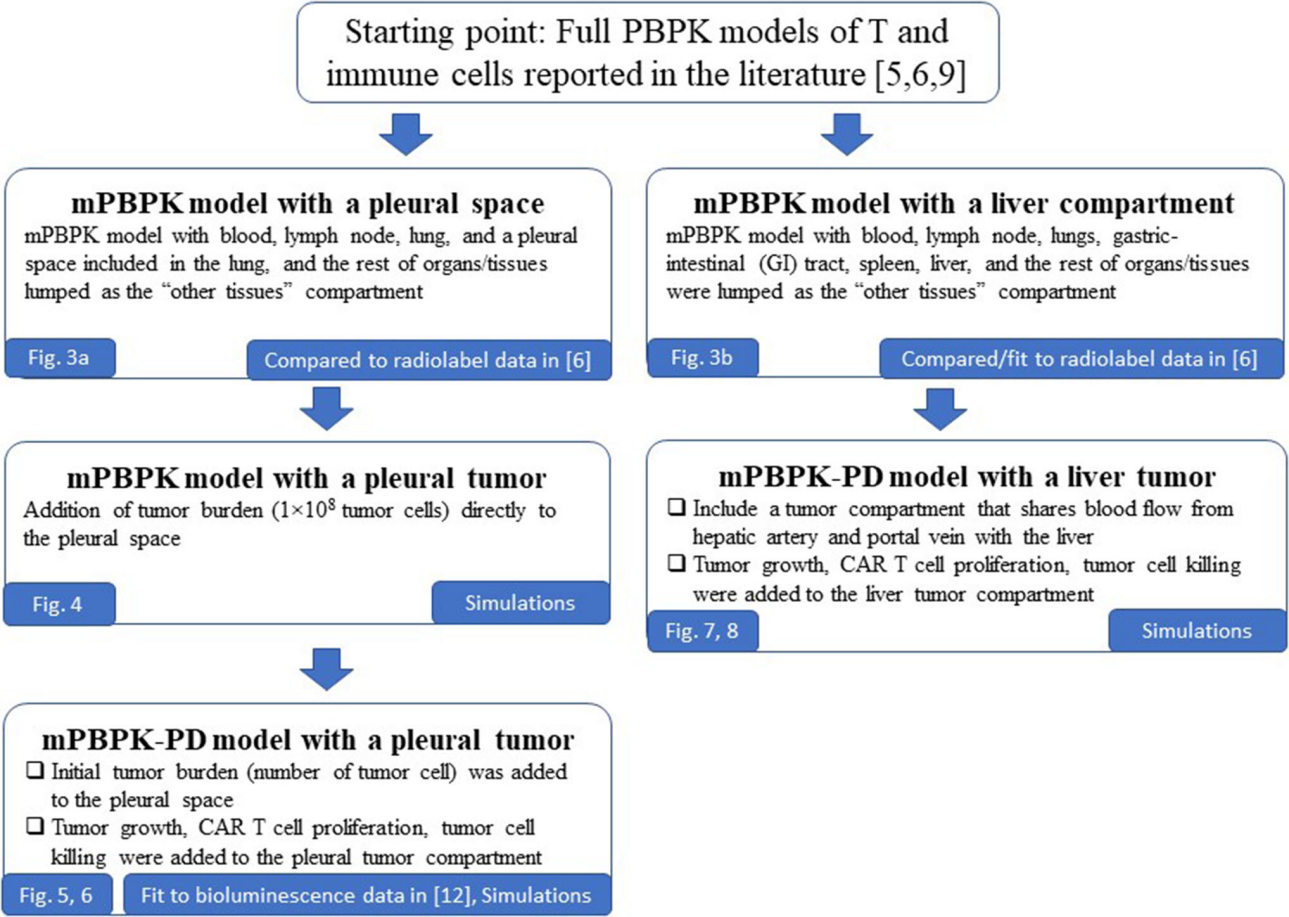
Workflow of the model development process

Minimal PBPK-PD models with a pleural or a liver tumor space were developed by adapting the reported full PBPK models of T cell distribution [5, 6, 9], followed by the addition of model structures representing a pleural or a liver tumor compartment. The model development workflow is outlined in Fig. 1. A comprehensive list of model equations and parameters of the mPBPK and mPBPK-PD models is provided in the Supplementary Materials under Model Equations. The model was developed in Phoenix WinNonlin (Build 8.1.0.3530, Certara, L.P., Princeton, NJ, USA). A proportional error model and a naïve-pooled algorithm were used for data fitting.

### Development of mPBPK models with pleural tumor space

**Fig. 2:**
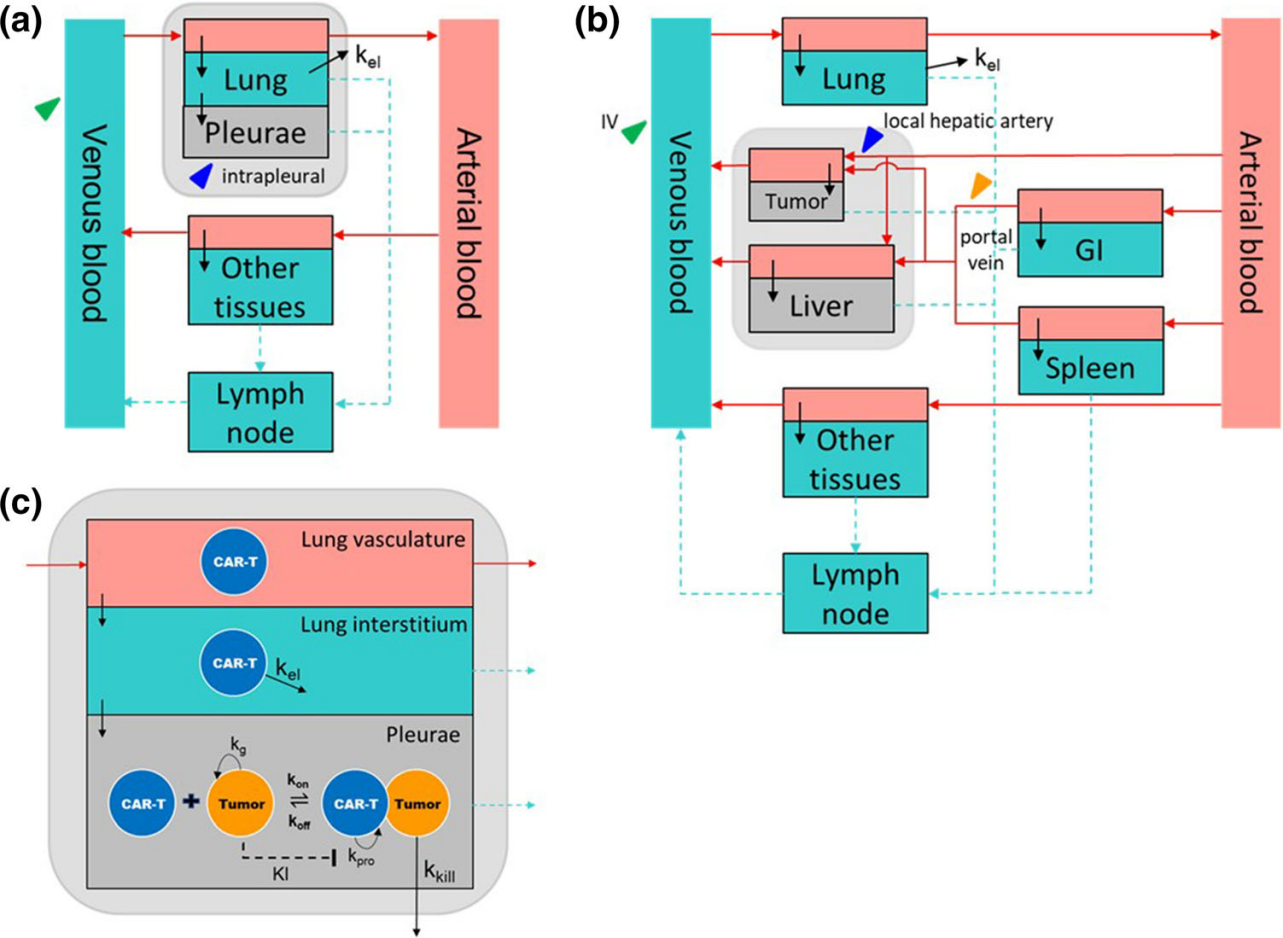
Schematics of model structures. **a** mPBPK model with a pleural space, **b** mPBPK model with a liver tumor, **c** PD components in the pleural space compartment

A mPBPK model was developed to represent explicitly venous blood, arterial blood, lungs, and lymph nodes tissues, while the rest of the organs/tissues were combined to form an “other tissues” compartment. The physiological parameters of the explicit compartments were obtained from the reported full PBPK models (Table S2), whereas the blood (*Q*_*ot*_) and lymph flow rates (*L*_*ot*_), and vascular (*Vv*_*ot*_) and interstitial volumes (*Vi*_*ot*_) of the “other tissue” compartment are the sum of respective parameters of each organ/tissue. The transmigration rate constant (*J*_*ot*_) of the “other tissues” was calculated using the equation,

1 $${J}_{ot}=\frac{\sum_{i}{J}_{i}\cdot {Vv}_{i}}{{Vv}_{ot, total}},$$

where *J*_*i*_ and *Vv*_*i*_ are the transmigration rate constant and vasculature volume of each organ/tissue, respectively, and *Vv*_*ot,total*_ is the combined vasculature volume of all organs/tissues in the “other tissue” compartment. The elimination of T cells was assumed to be in the lung compartment (*k*_*eli*_). The model subsequently added a pleural space in the lungs compartment (Fig. 2a). Because the physiological parameters of the pleural space in mice were not available in the literature, we assumed i) mouse pleural space volume is the same as that in human (0.26 mL/kg) after body weight normalization [10]; ii) the proportion of mouse pleural lymph flow rate is the same as that in dogs (2%) [11]. Lastly, the transmigration rate (*J*_*ps*_) to the pleural space was estimated from anti-mesothelin CAR T cellular kinetic profile in a mouse pleural tumor model [12] using the pleural space mPBPK-PD model described in the later section. Sensitivity analysis of mouse pleural space volume, pleural lymph flow rate and the transmigration rate was conducted to understand the impact of parameters on the T cell distribution in the blood, lungs and pleural space (Figs. S1–S3). To create a pleural tumor, tumor cells (initial tumor burden) was added directly to the pleural space.

### Development of mPBPK models with a liver compartment

A mPBPK model with a liver compartment was developed by explicitly representing the organs/tissues of interests, including blood, lungs, gastric-intestinal (GI) tract, spleen, liver, and lymph nodes, to describe the physiology of blood flow to the liver. The rest of the organs/tissues were combined to form the “other tissues” compartment, similar to the PBPK model described above. The physiological parameters of the explicitly represented compartments were obtained from the reported full PBPK models (Table S3), whereas the parameters of the “other tissue” compartment are the sum of respective parameters of each organ/tissue enrolled in the “other tissue” compartment. The transmigration rate constant (*J*_*ot*_) of the “other tissues” was calculated using Eq. (1) and, later on, was estimated by simultaneous fitting of reported T cell concentration profiles in blood, lungs, gastric-intestinal (GI) tract, spleen, liver (digitized from Khot et al. [6]) to the mPBPK model with the liver compartment developed herein.

### Development of mPBPK models with a liver tumor

A minimal PBPK model with a liver tumor was developed to imitate clinical CAR T hepatic artery delivery procedure for liver metastases [13]. As described by Katz et al. prior to the CAR T infusion, a specific hepatic artery branch that went into the patient liver tumor was mapped out using angiogram. CAR T was subsequently infused to the same artery branch by a pressure-enabled drug delivery device (PEDD™) to ensure complete delivery to the local tumor [13]. The clinical procedure led us to develop the mPBPK model with a liver tumor compartment under the following assumptions (Fig. 2b). First, the liver tumor was assumed to have the same anatomical features as the liver, so that the composition, i.e., percentage of vascular and interstitial volume, cell transmigration rate, and retention factor were the same as the liver. Secondly, the liver blood flow diverted to the tumor in the nominal case was ~ 10%, matching with the 10% of the initial tumor size (2.5 × 10^8^ cells, ~ 0.2 mL [9]). The conversion of the initial tumor size of 2.5 × 10^8^ cells to the tumor volume of ~ 0.2 mL was described in the supplementary materials. Lastly, CAR T cell was delivered through the diverted liver blood flow solely to the tumor. Although the assumptions oversimplify irregular tumor vasculature and high interstitial pressure observed in solid tumors that may impact on CAR T infiltration, the simplified tumor compartment allows us to isolate certain tumor parameters, such as tumor blood flow and transmigration rate constants, and evaluate their contribution to CAR T local tumor concentration in a systematic manner.

### Development of pharmacodynamic components in the pleural and liver tumor compartments

Tumor growth, CAR T cell proliferation, and tumor cell killing were added to the mPBPK models to form mPBPK-PD models. For tumor growth, an exponential growth model was assumed [14] and CAR T cell proliferation and tumor cell killing were accounted for by describing the interactions between the CAR and the TAA using macroscopic association and dissociation rate constants *k*_*on,mac*_ and *k*_*off,mac*_, respectively (Fig. 2c). These macroscopic rate constants aimed to describe cell-level binding interactions, which were a result of not only the bi-molecular binding *k*_*on*_ and *k*_*off*_, between the CAR and TAA, but also the cell diffusion and the avidity effect through multiple CAR-TAA pairs. The *k*_*on,mac*_ was fixed at 1 × 10^6^ M^−1^ s^−1^ during the parameter estimation because *k*_*on,mac*_ and *k*_*off,mac*_ are correlated and thus cannot be individually identified, and also because *k*_*on,mac*_ is mostly a diffusion limited parameter for cell–cell interaction [15]. The interactions between CAR and TAA formed an artificial immunological synapse that led to subsequent CAR T cell proliferation and tumor cell killing. CAR T cell proliferation was described by an exponential growth rate (*k*_*pro*_ $\times \left[\text{CAR T and tumor cell complex}\right]$) that was modulated by a tumor cell (TB) dependent inhibitory Hill function [16]. The mathematical term describing CAR T cell proliferation is therefore:

2 $${k}_{pro}\cdot \frac{1}{1+\frac{TB}{KI}}\cdot [\text{CAR T and tumor cell complex}]$$

where *k*_*pro*_ is the maximal CAR T cell proliferation rate, $[\text{CAR T and tumor cell complex}]$ is the number of CAR T and tumor cell complex joined through an artificial immunological synapse, *TB* is the total number of tumor cells, *KI* is the number of tumor cells needed to reduce CAR T cell proliferation rate by 50%.

Tumor cell killing was described by a first-order rate constant, *k*_*kill*_. The rate of tumor cell killing was therefore proportional to the number of CAR T and tumor cell pair. After a bound tumor cell was killed by a CAR T cell, the CAR T cell was released and became a free CAR T cell to continue the killing of tumor cells. No time delay in CAR T cell proliferation and tumor cell killing was assumed. Parameter values of the PD components in the pleural tumor model (*J*_*PS*_*, R*_*PS*_*, k*_*pro*_*, KI, k*_*kill*_*, k*_*off,mac*_; see Table 1 for the descriptions) were estimated by fitting to the digitized data [12] as described in the next section. PD parameter values (*k*_*pro*_*, KI, k*_*off,mac*_) in the liver tumor model used the same values derived from the pleural model. However, using the estimated pleural tumor *k*_*kill*_ in the liver model would require higher dose levels that exceed commonly used doses in preclinical tumor model studies in order to achieve efficacy. As the liver tumor model was intended for theoretical investigation and a previous study [8] reported more than tenfold span of k_kill_ across different CAR T therapies, the liver tumor *k*_*kill*_ was assumed to be twofold higher than the estimated pleural tumor *k*_*kill*_ in order to evaluate common dose levels (0.3–3 million cells/dose) reported in the literature.

**Table 1:** Fitted parameter values in mPBPK-PD model describing anti-mesothelin CAR T cell in a pleural tumor mouse model

Parameter	Description	Estimate	%CV	Unit
J_PS_	Rate of CAR T cell transmigration into the pleural space	0.119	25.6	1/h
R_PS_	Retention factor of CAR T cell in the pleural space	2.88	2.60	–
k_g_	Tumor growth rate	0.00385	8.19	1/h
k_pro_	Maximum CAR T cell proliferation rate	0.115	14.9	1/h
KI	Number of tumor cells required to inhibit 50% k_pro_	3.84 × 10^7^	15.4	Cells
k_kill_	Killing rate of tumor cells by CAR T cells	0.0733	3.60	1/h
k_off, mac_	Cell-level macroscopic dissociation rate constant	6.85 × 10^–8^	3.74	1/h

For the full list of parameters and their values, please refer to the supplementary materials

*CV* coefficient of variation, *RSE* relative standard error, %CV = %RSE

### Parameter estimation for the mPBPK-PD model with a pleural tumor

Bioluminescence data of anti-mesothelin CAR T cells and tumor cells in a pleural tumor mouse model [12] were digitized. The tumor growth rate (*k*_*g*_) was estimated using digitized control group data [12], and a doubling time of 7.5 days was obtained (Table 1). Next, the CAR T cell and tumor cell bioluminescence data with i.v. and intrapleural administration were simultaneously fitted to the pleural tumor mPBPK-PD model to estimate the PK parameters, *J*_*PS*_ and *R*_*PS*_, which describes the cell transmigration rate into the pleurae and the retention factor of CAR T cells in the pleurae, respectively, and the PD parameters, *k*_*pro*_*, KI, k*_*kill*_*, k*_*off,mac*_, where *k*_*pro*_ is the maximum proliferation rate of CAR T cells, *KI* is the number of tumor cells required to inhibit 50% of CAR T proliferation, *k*_*kill*_*,*is the maximum tumor killing rate by CAR T, and *k*_*off,mac*_ is the cell-level macroscopic association rate constant. The conversion of CAR T and tumor cell bioluminescence data to the absolute cell numbers were expressed in the following two equations:

3 $${BLI}_{CAR T}={S}_{1}\times {CAR T}_{total}$$

4 $${BLI}_{Tumor}={S}_{2}\times TB+{B}_{2}$$

where *BLI*_*CAR T*_ was the bioluminescence intensity of total CAR T cells in a mouse, S_1_ the scaling factor for CAR T cell signal, *BLI*_*Tumor*_ the bioluminescence intensity of tumor cells, S_2_ the scaling factor for tumor cell signal, B_2_ the baseline bioluminescence noise for tumor cells. The digitized data of tumor BLI at time zero had a range of fivefold. As initial tumor size was shown previously to be a sensitive parameter of the PK/PD responses [8], the geometric means of the digitized tumor BLI signals were used as the initial estimates for the number of tumor cells at time 0 (TB_0_) in the model. These parameters involved in the fitting process were included in Table S4.

## Results

### Minimal PBPK model with a pleural space

**Fig. 3:**
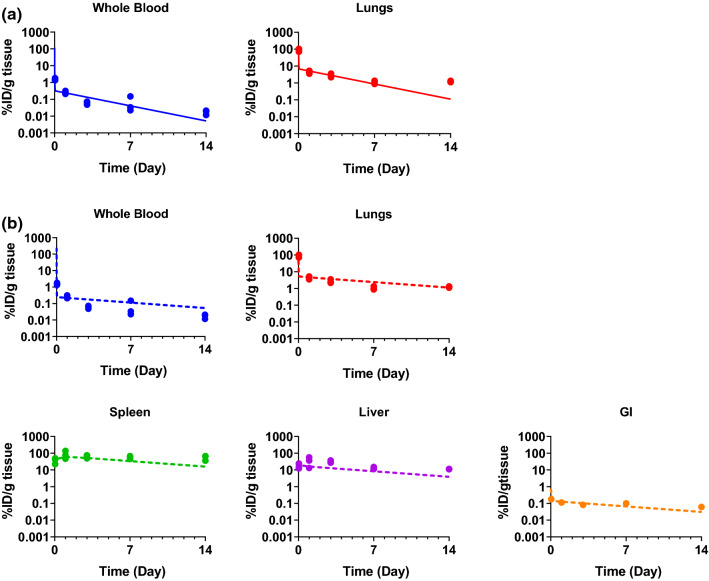
**a** Overlay of observed and mPBPK model simulated profiles of radiolabeled exogenous human T cells distribution in mouse whole blood and the lungs following i.v. administration. **b** Simultaneous fitting of mPBPK model to radiolabeled data of exogenous human T cells distribution in mouse whole blood, the lungs, spleen, liver and GI tract following i.v. administration

The mPBPK model with a pleural space was established to describe the initial distribution of exogenously administered human T cells in blood and lungs. The simulated T cell kinetics in whole blood and the lungs following i.v. administration are overlaid with the reported concentration profiles of radiolabeled exogenous T cells by Khot et al. [6]. As shown in Fig. 3a, the good agreement between the simulated and the digitized data suggests the mPBPK model with a pleural space well describes T cell distribution in the blood and the lungs.

### CAR T cell distribution following intrapleural injection

**Fig. 4:**
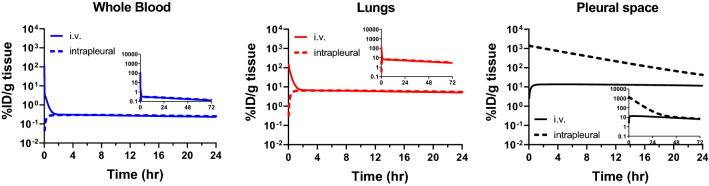
Simulation of exogenously administered non-binding T cell distribution in mice following i.v. (solid lines) or intrapleural administration (dashed lines) using the mPBPK model with a pleural space and assuming a fixed tumor burden of 1 × 10^8^ tumor cells. Insets are CK profiles from 0 to 72 h

The established mPBPK model with a pleural space was subsequently used to explore the benefit of intrapleural administration in local CAR T cell exposure by introducing 1 × 10^8^ tumor cells to the pleural space and administering non-binding T cells through i.v. or local delivery. As shown in Fig. 4, transient difference in the cellular kinetic profiles following i.v. and intrapleural administration is apparent in the first 4 h (h). However, the cellular kinetics difference diminished within 72 h, as the profiles shown in the insets of Fig. 4 all overlap after 72 h. The difference is further quantified by the AUC in the organs/tissues over the study duration (Table 2). The exposure in the pleural space in the first 4 h after intrapleural administration is approximately 80-fold higher than that after i.v. administration, whereas the exposures in blood and the lungs are 5- and threefold lower than that of i.v. administration, respectively, suggesting a synchronized benefit of increasing exposure at the site of interest while decreasing exposure in organs/tissues where CAR T cells distributed are not desired. However, by 72 h, the difference in AUC of the pleurae decreases to approximately 13-fold, while the AUCs in blood and the lungs become comparable.

**Table 2:** Predicted exposure by mPBPK model following i.v. or intrapleural administration of non-binding cells in the case of fixed tumor burden of 1 × 10^8^ tumor cells

Organ	AUC_0-4 h_ (%ID·h/g tissue)		${\left.\frac{{AUC}_{intrapleural}}{{AUC}_{i.v.}}\right|}_{0-4 \,h}$	AUC_0-72 h_ (%ID·h/g tissue)		${\left.\frac{{AUC}_{intrapleural}}{{AUC}_{i.v.}}\right|}_{0-72 \, h}$
	i.v	Intrapleural		i.v	Intrapleural	
Blood	5.33	1.09	0.205	19.1	15.9	0.832
Lungs	62.1	23.6	0.380	364	347	0.953
Pleural space	50.4	4130	81.9	728	9460	13.0

As the physiological parameters of mouse pleural space were estimated from literature data extrapolation described in the Methods, sensitivity analysis was conducted to understand the impact of volume of pleural cavity (Vi_PS_), lymph flow rate in the pleural space (L_PS_), and transmigration rate from the lungs to the pleural space (J_PS_) on the CAR T cell concentration in the pleural space. A range of 25-fold was studied for Vi_PS_ and L_PS_, while 100-fold was studied for J_PS_. With i.v. administration (Figs. S1a, S2a, S3a), the cellular kinetic profiles in blood and the lungs were not sensitive to any of the 3 parameters, whereas the cellular kinetic profile in the pleural space was highly sensitive to J_PS_, apparently because J_PS_ determines the rate of cells transmigrate from the lungs to the pleural space. In comparison, Vi_PS_ and J_PS_ were not sensitive parameters with intrapleural administration (Figs. S1b, S2b, S3b), while a slower lymph flow rate (lower L_PS_) was able to significantly increase CAR T cell level in the pleural space and reduce the level in blood and the lungs. Nonetheless, the sensitivity analysis further supports the finding of initial and transient higher concentration of CAR T in the pleural space following intrapleural administration.

### Minimal PBPK-PD model fitting of pleural tumor model

**Fig. 5:**
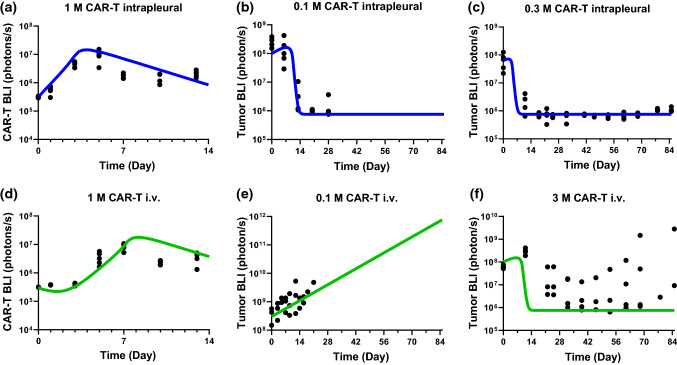
Minimal PBPK/PD modeling of digitized bioluminescence data (black connected dots) of anti-mesothelin CAR T cells and tumor cells in mice bearing a pleural tumor. CAR T dose levels range from 0.1 × 10^6^ (0.1 M) to 3 × 10^6^ (3 M) cells. **a**–**c** i.v. administration (green lines); **d**–**f** intrapleural administration (blue lines) (Color figure online)

To understand efficacy benefit of transient higher concentration of CAR T cell in pleural space following intrapleural injection, a pleural tumor model was added to the base model to describe literature reported mesothelin-targeting CAR T cellular kinetics and the tumor growth inhibition profiles following i.v. and intrapleural delivery [12] (Fig. 5). At 1 × 10^6^ CAR T cell dose, there is immediate CAR T cell expansion following intrapleural administration (Fig. 5a), whereas the expansion of CAR T cells starts ~ 2 days later following i.v. administration (Fig. 5d). Although the peak CAR T cell signals (Cmax) are comparable between i.v. and intrapleural administration, the time to Cmax, Tmax, shows consistently a 2-day delay for the i.v. treatment group compared to intrapleural administration. More interestingly, tumor growth following 0.1 × 10^6^ cells i.v. and intrapleural administration show drastically different responses, where intrapleural administration (Fig. 5b) leads to tumor regression on Day 7 and onward, while i.v. administration (Fig. 5e) does not show efficacy. At 0.3 × 10^6^ cells intrapleural dose (Fig. 5c), tumor regression is sustained up to Day 85. In contrast, despite a tenfold increase in dose, mice treated with an i.v. dose of 3 × 10^6^ cells show variable responses, where, after initial tumor regression observed after Day 14, the tumor grows back to various degrees after Day 42 (Fig. 5f). The mPBPK-PD model (blue and green lines) is shown to reasonably capture most of the trends in the digitized data, except for the recurrence of tumor after initial tumor growth inhibition at 3 × 10^6^ cells i.v. dose. To improve the fitting at the i.v. high dose, a Hill function and a self-inhibition function (a bell shape response) were introduced to the *k*_*kill*_. However, these additional parameters failed to simultaneously describe all intrapleural and i.v. profiles to equal satisfaction. It is hypothesized that delayed CAR T expansion following i.v. dosing may be more prone to exhaustion, therefore, a time-dependent *k*_*kill*_ may be more appropriate to describe the diminishing killing effect. However, a first order time-dependent *k*_*kill*_, i. e. *k*_*kill*_·*e*^*−kt*^, was still unable to fully capture the profiles. It is likely that the time-dependent *k*_*kill*_ is driven by multiple factors, such as the dosing route and the timing of CAR T activation. As a result, a linear *k*_*kill*_ is kept in the mPBPK-PD model due to the lack of pharmacodynamic data in the literature [12]. The estimated parameter values were summarized in Table 1.

**Fig. 6:**
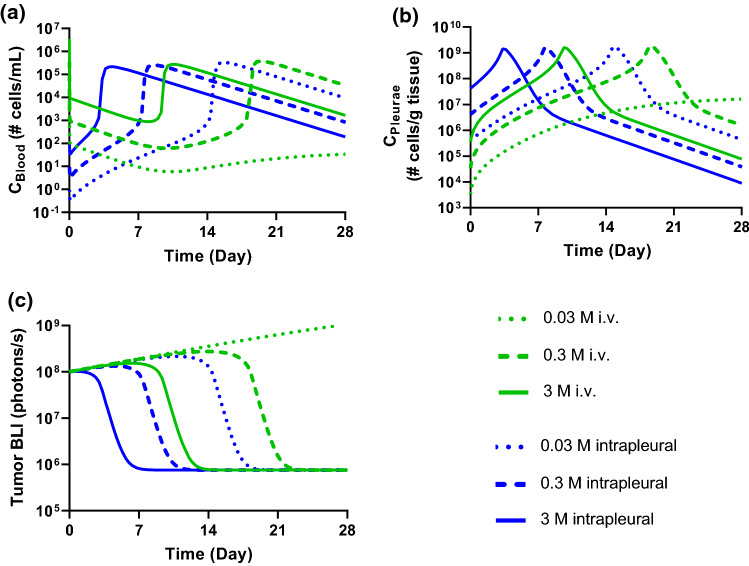
Theoretical investigation of dose-dependent and dosing route-dependent cellular kinetics and tumor growth inhibition using the fitted mPBPK-PD model with a pleural tumor. Simulated dose levels: 0.03 × 10^6^ (0.03 M), 0.3 × 10^6^ (0.3 M), and 3 × 10^6^ (3 M) cells. **a** CAR T cell concentration in blood. **b** CAR T cell concentration in tumor. **c** Tumor growth profiles

To further explore the dose-dependent cellular kinetics and tumor growth inhibition relationship, we used the calibrated mPBPK-PD model to simulate CAR T cellular kinetic and efficacy profiles in mice with initial pleural tumor burden of 1 × 10^8^ tumor cells. Three CAR T cell doses, 0.03, 0.3, and 3 × 10^6^ cells/mouse following both i.v. and intrapleural administration were evaluated. (Fig. 6). The simulated CAR-T cell kinetics and tumor BLI revealed several interesting aspects: (i) at low dose (0.03 × 10^6^ cells/mouse), CAR T expansion and tumor regression occurred following intrapleural administration, but not following i.v. administration; while at middle and high doses (0.3 and 3 × 10^6^ cells), intrapleural dosing is associated with earlier CAR T cell expansion and earlier tumor regression comparing at the same i.v. doses; (ii) Cmax after CAR T expansion appears to be similar among three dose groups, whereas Tmax shifts to an earlier time point at higher doses, suggesting that Tmax, but not Cmax, is dependent on both the dosing routes and the dose levels; (iii) The CAR T cell concentration in blood and tumor have similar time to peak profile, indicating that CAR T concentration in the blood may be a reliable surrogate for its tumor concentration (Fig. 6a and b).

### Minimal PBPK model with a liver compartment

**Fig. 7:**
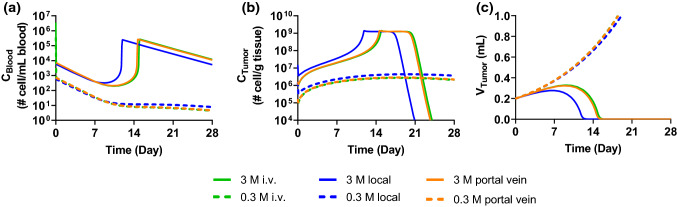
Theoretical investigation of the benefits of local administration: mPBPK-PD model with a liver tumor is used for simulating the **a** blood cellular kinetics, **b** CAR T cell infiltration in tumor, and **c** efficacy profiles as a result of CAR T cell administration through local hepatic artery administration, i.v. administration, and portal vein administration at dose levels of 0.3 × 10^6^ (0.3 M) and 3 × 10^6^ (3 M) CAR T cells

**Fig. 8:**
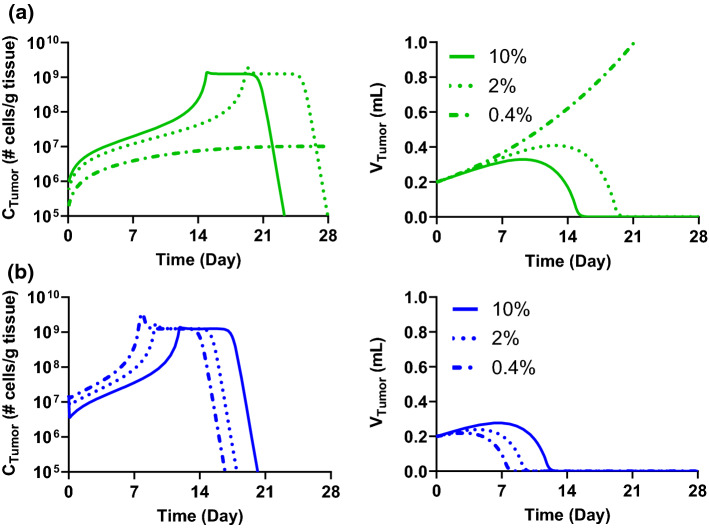
Theoretical simulation of the impact of volumetric blood flow rate in tumor on cellular kinetics and tumor growth inhibition using the mPBPK-PD model representing a liver tumor following a single dose of 3 × 10^6^ (3 M) cells through **a** i.v. or **b** local delivery. 10%, 2%, and 0.4% represent the approximate percentage of tumor blood flow rate compared to total liver flow rate (liver tumor tissue plus normal tumor tissue)

The mPBPK model with a liver compartment was established to describe the initial distribution of exogenously administered human T cells in mice. Simulations of the T cell concentration profiles in whole blood, lungs, spleen, liver, and GI were overlaid with the digitized dataset from the same literature used in the pleural mPBPK model development [6] (Fig. S4). However, the simulated profiles appeared to underestimate concentrations in liver, spleen, and GI tract, which could be attributed to a less representative transmigration rate constant (*J*_*ot*_, 16.7 1/h) calculated using Eq. (1). To address this, the mPBPK model with a liver compartment was used to fit the digitized data [6] (Fig. 3b) to derive a fitted *J*_*ot*_ (94.3 1/h, Table S3). Later, simulations of cellular kinetic and efficacy profiles using the established liver tumor mPBPK-PD model yielded very similar profiles (Figs. 7, 8 vs. Figs. S5–S8), when either the Eq. (1) calculated or the fitted *J*_*ot*_ was used, suggesting that *J*_*ot*_ is not a sensitive parameter in the mPBPK-PD model.

### Minimal PBPK-PD model simulation of liver tumor model

Hepatic artery infusion of CEA-targeting CAR T cells has been evaluated in clinical trials for treating liver metastases [13, 17]. Preclinical evaluation of hepatic artery delivery of the CAR T cells in mouse liver metastases tumor model, however, is not feasible due to surgical challenges. As a result, portal vein administration has been considered as an alternative approach for hepatic local delivery in preclinical settings [18]. Nevertheless, the portal vein blood flow, which accounts for 25% cardiac output, is much faster than tumor local hepatic artery blood flow, and only a fraction of the portal vein blood flow reaches the local liver tumor. Whether portal vein dosing emulates hepatic artery administration was tested by the mPBPK-PD model with a liver tumor.

In the liver tumor mPBPK-PD model (Fig. 2b), the initial liver tumor size (TB_0_) was assumed to be 2.5 × 10^8^ cells, corresponding to ~ 0.2 mL tumor volume or 10% of mouse liver volume. In addition, the TB_0_ of 2.5 × 10^8^ cells was within the TB_0_ range in the previous pleural mPBPK-PD model, that allowed us to adopt the PD parameters derived from the pleural tumor model for conceptual cellular kinetics and PD simulation (Table S3). In Fig. 2b, local tumor delivery of CAR T cells (blue arrowhead) assumes that CAR T cells are administered through a branch of hepatic artery that supplies the blood entirely to the tumor. This assumption is supported by the clinical procedure of hepatic artery infusion to maximize CAR T delivery to the local tumor as described in the Methods [13]. As the assumed initial tumor size is ~ 10% of liver volume, the blood flow at local tumor injection site is assumed to be 18 mL/h, approximately 10% of total hepatic artery blood flow (18.7 mL/h) plus portal vein blood flow (151.88 mL/h, Q_GI_ + Q_Spleen_ − L_GI_ − L_Spleen_) (Table S3) [9]. Portal vein injection (orange arrowhead) assumes that a fraction of the cells goes into the liver tumor vasculature and the rest goes to the normal liver vasculature according to the blood flow rates into the respective compartments. Intravenous administration (green arrowhead) was also simulated to examine the benefit of local administration of cells through either local tumor delivery or portal vein injection.

The simulated blood cellular kinetics and tumor growth inhibition profiles following portal vein and i.v. administration are largely similar, with nearly overlapping cellular kinetics and efficacy profiles at both dose levels, suggesting limited benefit of portal vein injection in mice compared to i.v. administration (Fig. 7). In contrast, local tumor delivery results in much higher concentration of CAR T cells in the tumor compartment (Fig. S7), which leads to earlier CAR T cell proliferation that correlates with earlier tumor growth inhibition (Fig. 7), similar to the findings following intrapleural injection. The simulation of liver tumor model suggests that the benefit of local tumor delivery can be attributed to the first pass process as the higher CAR T cell concentration in the tumor compartment following local delivery diminishes afterwards. Moreover, as 10% of total hepatic blood flow is assigned to the liver tumor compartment in the simulation, portal vein injection delivers only 10% CAR T cells to the tumor during the first pass process, with the rest 90% CAR T cells enter into systemic circulation almost immediately, which leads to negligible difference in C_Tumor_ following portal vein and i.v. dosing. Conversely, a much slower blood flow rate at local tumor artery site not only delivers 100% CAR T to the local tumor compartment, the slower blood flow also allows CAR T cell longer time to transmigrate to tumor interstitium before entering into systemic circulation, thus, leading to more pronounced benefit in CAR T delivery. In summary, the mPBPK-PD simulation suggests portal vein injection in preclinical mouse tumor model may underestimate the benefit of local tumor hepatic artery delivery in patients.

Next, we examined the impact of different tumor volumetric blood flow rates on CAR T cell delivery through i.v. and local hepatic artery administration (Fig. 8a and b). In the simulation, the tumor volumetric blood flow rate was reduced to 5- or 25-fold lower than the nominal flow rate (~ 10% of total liver blood flow). With i.v. administration (Fig. 8a), as the tumor volumetric blood flow rate decreases, a lower C_Tumor_ is observed in the simulated profiles. Thus, higher volumetric blood flow rate into the tumor helps the cells infiltrate into the tumor and subsequent anti-tumor efficacy. On the other hand, with local tumor delivery, a decrease in tumor volumetric blood flow leads to an increase in initial CAR T cell tumor distribution (Fig. 8b). This is expected since the CAR T cells are directly delivered into the tumor vasculature, and a lower volumetric blood flow rate would indicate a longer residence time in tumor vasculature, thereby increasing the chance of CAR T cells transmigrating into tumor interstitium. Even though the simulation of local tumor delivery suggests substantial CAR T cell proliferation and tumor growth inhibition irrespective of the tumor blood flow rates, it is possible that, for less potent CAR T cells, the effect of blood flow rate would lead to more distinct cellular kinetics and efficacy profiles.

## Discussion

The field of cell therapy for cancer treatment has gained tremendous momentum as manifested by recent approvals of CD19- and BCMA-targeting autologous CAR T cell therapies and emerging novel allogeneic CAR T and NK cell platforms for solid tumor treatment. Challenges facing CAR T cell therapies include tumor antigen loss, biological barriers against CAR T cell infiltration, expansion, and persistence, and dysfunction of CAR T cells that leads to toxicity [19]. As these challenges often are reflected in the CAR T cellular kinetic profiles [20], and are monitored in the clinic to correlate with efficacy and safety, mechanistic understanding of CAR T cellular kinetic behavior has gradually been accumulated [19, 20]. However, CAR T cellular kinetics is usually not thoroughly examined in the preclinical phase, rendering the difficulties of cellular kinetics translation from preclinical species to humans. Limited nonclinical cellular kinetics data reported in the literature have shown that i.v. administration of anti-mesothelin and anti-BCMA CAR T cells in tumor bearing NSG mice would lead to a Tmax ranging from 5 to 14 days. [12, 21, 22], which is comparable to the human Tmax of 7–14 days reported for i.v. administration of anti-CD19 and anti-disialoganglioside (GD2) CAR T cells [3, 4, 23–25]. The implication of such observation is two-fold: i) the time scale of CAR T cell expansion, if present, is comparable between human and mouse, presumably due to similar interactions between the human CAR T cells and human tumor cells in the two species; ii) if the CAR T cells can infiltrate into a solid tumor and remain functional, the cellular kinetics and PD responses may have similar features compared to the responses observed with liquid tumor.

The mPBPK-PD models in this study were developed based on published models [5, 6, 9] with an emphasis on cellular kinetics-PD-efficacy relationship after local CAR T delivery. The major assumptions here are: (i) CAR T cell elimination occurs in the lungs [5, 6]; (ii) expression of TAA is completely restricted to the tumor cells so the interaction between CAR and TAA only occurs in the tumor compartment; (iii) the killing of tumor cells is solely driven by the CAR T-tumor cell pair, and the effects of the immunosuppressive TME and other cytokines are not considered; (iv) the CAR T cells are treated as one single population, so effector and memory T cells are not distinguished in the model; (v) no time delay between formation of the CAR T and tumor cell complex and tumor cell killing; (vi) CAR T cells remain active over the study duration. No CAR T cell exhaustion is considered. Although these assumptions oversimplify CAR T in vivo disposition and mechanism of action, the models serve the purpose for evaluating the impact of dosing routes on CAR T distribution while maintaining the flexibility of building in more detailed mechanistic modules once experimental data become available from future characterization.

The mPBPK portion of the model was used for investigating the benefit of intrapleural delivery, and it was shown that difference in CAR T cell exposure following different dosing routes would be transient and diminish within 72 h (Fig. 4). With intrapleural administration, simultaneous increase in exposure at the site of interest (pleurae) and decrease in exposure in all other organs/tissues could provide potential benefit in safety. As the mPBPK model assumed no expression of TAA in any of the organs/tissues, it is possible that the presence of TAA at the site of interest could further improve the exposure with local delivery as CAR T cells could interact with TAA-expressing cells (presumably tumor cells) and stay at the site of interest, thereby extending the benefit of local delivery.

The mPBPK-PD model with a pleural tumor captured cellular kinetic profiles from a mouse pleural tumor model treated with anti-mesothelin CAR T cells through i.v. or intrapleural administration. In Fig. 5d, the cellular kinetic profile following i.v. dosing showed initial delay followed by rapid expansion and subsequent slow contraction, consistent with typical CAR T cellular kinetic profiles [21]. In comparison, intrapleural injection demonstrated similar cellular kinetic profiles but with a much earlier CAR T expansion (Fig. 5a). Further examination of the cellular kinetic profiles in the pleurae (Fig. 6b) showed clear benefits of local delivery at the low dose level (0.03 × 10^6^ cells), where CAR T expansion and tumor regression achieved following intrapleural administration, but not following i.v. administration at the same dose. It indicates that a threshold C_Tumor_ must be reached before CAR T cell expansion and substantial tumor cell killing can occur (Fig. 6a–c). If a certain type of solid tumor is more resistant to CAR T cell infiltration, the i.v. dose required to reach the threshold C_Tumor_ could be unattainable due to manufacture limitations or safety concerns. Local delivery can thus be a potential solution for overcoming such dose limitation. On the other hand, the pleural mPBPK-PD model and the digitized data suggest a difference of at least 30-fold in efficacy between intrapleural and i.v. delivery (0.1 × 10^6^ cells for intrapleural delivery and 3 × 10^6^ cells for i.v. delivery). It is likely that such fold-difference between local delivery and systemic administration would be dependent on cancer indications and CAR T cell products. As local delivery is typically associated with substantial technical challenges, the benefits of local delivery compared to systematic administration will need to be evaluated carefully for each CAR T cell therapy [8, 26].

The mPBPK-PD model with a liver tumor revealed a critical aspect of local hepatic artery administration: cells need to be given to the direct blood supply source of the tumor in order to maximize the benefit of local administration (Fig. 7). The simulations using the liver tumor mPBPK-PD model further suggested the potential of local delivery when a tumor is poorly vascularized. Lower volumetric blood flow rate should be expected in such tumor and the threshold C_Tumor_ would therefore be harder to reach through i.v. administration, whereas the lower volumetric blood flow rate would work towards the benefits of locally delivered CAR T cells (Fig. 8). As volumetric blood flow rate is only one of the attributes associated with solid tumor, other attributes such as lymph flow rate, vasculature volume, interstitial volume, and immune suppressive microenvironment, etc., could also play a substantial role which may lead to high inter-subject variabilities in cellular kinetics and efficacy of CAR T cell therapies. Further investigation will be needed to elucidate the significance of these different aspects.

One limitation of the mPBPK-PD models is the assumption of a homogenous CAR T population with the same anti-tumor activity over the study duration. This assumption, however, cannot be true because CAR T is a living drug. It has been shown that the composition of CAR T cells, e.g. CD4+, CD8+ effector and memory cells, as well as the T cell activation and exhaustion phenotypes, evolves over time [12, 23, 27]. Despite that the time-dependent heterogeneity of CAR T cell population plays an important role in the outcome of CAR T cell therapies [12, 23, 27], the phenotypic (i.e. activity) change of CAR T cells cannot be easily monitored, for example, by measuring the CAR T cell concentrations. The lack of information on CAR T activity over time lead to uncertainties in the cellular kinetics and efficacy relationship. Indeed, in Fig. 5f, the model fitted curve overestimated initial tumor regression rate and failed to capture the rebound of tumor growth after i.v. administration of 3 × 10^6^ cells. The overestimation is largely driven by the robust CAR T expansion observed after 1 × 10^6^ cells i.v. administration (Fig. 5d). It is possible that the delayed cell expansion after i.v. administration leads to a less active and persistent CAR T cell phenotype. In the literature where we obtained the cellular kinetics and efficacy data, it suggested that effector memory T cells were found to be contributing to the long term persistence of intrapleurally administered cells (Fig. 5 in [12]). Unfortunately, no immunophenotyping information was provided for the i.v. administration cases. Our modeling effort revealed the importance of characterizing CAR T cells fitness over the study duration, for example, using kinetic immunophenotyping, to better understand the cellular kinetics and the duration of response. Emerging efforts in quantitative characterization different phenotypes of CAR T cells have been reported in the literature [7, 28, 29].

While the mPBPK-PD models developed in this study are able to capture the improved biodistribution to tumor through local delivery (Figs. 5, 6, 7 and 8), the safety aspect of CAR T cell treatment [30] has not yet been considered in the current models. That local delivery generally leads to less toxicity in humans and mice (with one reported exception [31]) is still a phenomenon not well understood. In humans, local delivery of CAR T cells has shown to be safe, as no treatment-related adverse event greater than grade 3 has ever been reported for local delivery (Table S1). However, it is unclear how such safety feature is associated with or disconnected from the cell expansion phase, since most clinical cellular kinetics data for solid tumor treatment are not publicly available yet. As previous modeling effort [7] found that the magnitude of cytokine elevation is more related to baseline disease burden than the dose level, tumor growth dynamics could be a critical factor for understanding the rise of a better safety profile through local administration. Further experimental characterization of CAR T cell therapies is needed for additional mechanistic understanding of the improved safety profiles through local delivery.

## Conclusions

In this work, minimal PBPK-PD models were constructed to investigate the effect of dosing routes on CAR T cellular kinetics and efficacy in mouse tumor models. The mathematical model with a pleural space captured published cellular kinetics and tumor growth inhibition data of anti-mesothelin CAR T cells in a mouse model with a pleural tumor, indicating initial transient increase in exposure following local delivery contributed to improved anti-tumor efficacy comparing to systemic CAR T delivery. In addition, the mPBPK-PD model revealed a threshold of CAR T cells in tumor in order for their proliferation and efficacy. Local CAR T delivery elevated initial CAR T cell concentration to reach the threshold, thereby resulting in improved efficacy. Simulations using the established mPBPK-PD model with a liver tumor compartment suggest that the benefit of hepatic artery administration depends on volumetric blood flow rate at the injection site and the fraction of the blood flow delivered to the tumor. Higher volumetric blood flow rate to the tumor site is preferable for CAR T tumor distribution following i.v. administration, whereas lower volumetric blood flow rate at the hepatic artery injection site near the tumor is beneficial for CAR T distribution to the hepatic tumor. As current investigation brought insights into the contribution of blood flow to CAR T distribution to solid tumors, future effort will strive to understand migration behavior of heterogenous CAR T subsets and phenotypes and the impact on its safety and efficacy [32].

## Supplementary Information

Below is the link to the electronic supplementary material.

Supplementary file1 (DOCX 489 KB)
