# Supplementary material for: Development of minimal physiologically-based pharmacokinetic-pharmacodynamic models for characterizing cellular kinetics of CAR T cells following local deliveries in mice
Source: J Pharmacokinet Pharmacodyn. 2022 Jul 22;49(5):525–38. doi: 10.1007/s10928-022-09818-8 (PMC9508025; doi:10.1007/s10928-022-09818-8)
Supplement: Supplementary file 1 — Supplementary file1 (DOCX 489 KB) [file 10928_2022_9818_MOESM1_ESM.docx]

Supplementary Information

**Title:** Development of Minimal Physiologically-Based Pharmacokinetic-Pharmacodynamic Models for Characterizing Cellular Kinetics of CAR T Cells Following Local Deliveries in Mice

**Journal:** Journal of Pharmacokinetics and Pharmacodynamics

**Authors:** Chia-Hung Tsai, Aman P. Singh, Cindy Q. Xia, Haiqing Wang

**Title:** Development of Minimal Physiologically-Based Pharmacokinetic-Pharmacodynamic Models for Characterizing Cellular Kinetics of CAR T Cells Following Local Deliveries in Mice

**Affiliation:** Takeda Pharmaceutical Company Limited, Cambridge, MA, USA

**Contact information of corresponding author:**

Haiqing Wang

Email: haiqing.wang@takeda.com

Telephone: +1-857-600-3440

Postal address: 35 Landsdowne St, Cambridge, MA 02139, USA

List of Content in Supplementary Information

**Fig. S1** Sensitivity analysis with pleural space volume as the parameter of interest.

**Fig. S2** Sensitivity analysis with lymph flow rate in the pleural space as the parameter of interest.

**Fig. S3** Sensitivity analysis with transmigration rate to the pleural space as the parameter of interest.

**Fig. S4** Overlay of observed and mPBPK model simulated profiles of radiolabeled exogenous human T cells distribution in mouse whole blood, the lungs, spleen, liver and GI tract following i.v. administration.

**Fig. S5** Theoretical investigation of the benefits of local administration using mPBPK-PD model with a liver tumor and fitted parameters derived in Fig. 3b: (a) blood CK, (b) CAR T cell infiltration in tumor, and (c) efficacy profiles as a result of CAR T cell administration through local hepatic artery administration, i.v. administration, and portal vein administration at dose levels of 0.3×10^6^ (0.3 M) and 3×10^6^ (3 M) CAR T cells

**Fig. S6** Theoretical simulation of the impact of blood flow rate in tumor using the mPBPK-PD model representing a liver tumor with parameters derived from Fig. 3b. Dosing regimens investigated are single dose of 3×10^6^ (3 M) cells through (a) i.v. or (b) local delivery. 10%, 2%, and 0.4% represent the approximate percentage of tumor blood flow rate compared to total liver flow rate (liver tumor tissue plus normal tumor tissue)

**Fig. S7** Liver tumor mPBPK-PD model: Simulation of initial distribution of CAR T cells in (a) blood and (b) tumor following a single dose of 0.3×10^6^ (0.3 M) or 3×10^6^ (3 M) cells through i.v., local tumor, or portal vein administration

**Fig. S8** Liver tumor mPBPK-PD model with fitted parameters derived in Fig. 3b: Simulation of initial distribution of CAR T cells in (a) blood and (b) tumor following a single dose of 0.3×10^6^ (0.3 M) or 3×10^6^ (3 M) cells through i.v., local hepatic artery, or portal vein administration.

**Model equations** for mPBPK-PD model with i) pleural tumor; ii) liver tumor

**Table S1** Summary of CAR T cell therapies that have been investigated through non-systemic deliveries in mice and humans

**Table S2** Full list of parameters in mPBPKPD model for pleural tumor

**Table S3** Full list of parameters in mPBPKPD model for liver tumor

**Table S4** Parameters involved in fitting to pleural tumor data

**Fig. S1** Sensitivity analysis with pleural space volume as the parameter of interest

**Fig. S2** Sensitivity analysis with lymph flow rate in the pleural space as the parameter of interest

**Fig. S3** Sensitivity analysis with transmigration rate to the pleural space as the parameter of interest

**Fig. S4** Overlay of observed and mPBPK model simulated profiles of radiolabeled exogenous human T cells distribution in mouse whole blood, the lungs, spleen, liver and GI tract following i.v. administration

**Fig. S5** Theoretical investigation of the benefits of local administration using mPBPK-PD model with a liver tumor and fitted parameters derived in Fig. 3b: (a) blood CK, (b) CAR T cell infiltration in tumor, and (c) efficacy profiles as a result of CAR T cell administration through local hepatic artery administration, i.v. administration, and portal vein administration at dose levels of 0.3×10^6^ (0.3 M) and 3×10^6^ (3 M) CAR T cells

**Fig. S6** Theoretical simulation of the impact of blood flow rate in tumor using the mPBPK-PD model representing a liver tumor with parameters derived from Fig. 3b. Dosing regimens investigated are single dose of 3×10^6^ (3 M) cells through (a) i.v. or (b) local delivery. 10%, 2%, and 0.4% represent the approximate percentage of tumor blood flow rate compared to total liver flow rate (liver tumor tissue plus normal tumor tissue)

**Fig. S7** Liver tumor mPBPK-PD model: Simulation of initial distribution of CAR T cells in (a) blood and (b) tumor following a single dose of 0.3×10^6^ (0.3 M) or 3×10^6^ (3 M) cells through i.v., local tumor, or portal vein administration

**Fig. S8** Liver tumor mPBPK-PD model with fitted parameters derived in Fig. 3b: Simulation of initial distribution of CAR T cells in (a) blood and (b) tumor following a single dose of 0.3×10^6^ (0.3 M) or 3×10^6^ (3 M) cells through i.v., local hepatic artery, or portal vein administration.

Model Equations

i) Pleural tumor

**Compartment 1: Arterial blood**

$$V_{ab}\cdot\frac{dC_{ab}}{dt}=C_{Lungs,vas}\cdot Q_{Blood}-C_{ab}\cdot Q_{ot}$$

**Compartment 2: Venous blood**

$$V_{vb}\cdot\frac{dC_{vb}}{dt}=C_{ot,vas}\cdot\left( Q_{ot}-L_{ot} \right)+C_{LN}\cdot L_{total, lymph}-C_{vb}\cdot\left( Q_{Blood}+L_{Lungs} \right)$$

**Compartment 3: Lungs**

# vasculature

$${Vv}_{Lungs}\cdot\frac{dC_{Lungs,vas}}{dt}=C_{vb}\cdot\left( Q_{Blood}+L_{Lungs} \right)-C_{Lungs,vas}\cdot J_{Lungs}\cdot{Vv}_{Lungs}-C_{Lungs,vas}\cdot Q_{Blood}$$

# interstitium

$${Vi}_{Lungs}\cdot\frac{dC_{Lungs,int}}{dt}=C_{Lungs,vas}\cdot J_{Lungs}\cdot{Vv}_{Lungs}-C_{Lungs,int}\cdot\left( L_{Lungs}-L_{PS} \right)-k_{el}\cdot C_{Lungs,int}\cdot{Vi}_{Lungs}-C_{Lungs,int}\cdot J_{PS}\cdot{Vi}_{Lungs}$$

**Compartment 4: Other tissues**

J_ot_ is derived theoretically using the following equation:

$$J_{ot}=\frac{\sum_{i} J_{i}\cdot{Vv}_{i}}{{Vv}_{ot, total}}$$

where i indicates the organs to be lumped into the other tissues compartment.

# vasculature

$${Vv}_{ot}\cdot\frac{dC_{ot,vas}}{dt}=C_{ab}\cdot Q_{ot}-C_{ot,vas}\cdot J_{ot}\cdot{Vv}_{ot}-C_{ot,vas}\cdot\left( Q_{ot}-L_{ot} \right)$$

# interstitium

$${Vi}_{ot}\cdot\frac{dC_{ot,int}}{dt}=C_{ot,vas}\cdot J_{ot}\cdot{Vv}_{ot}-C_{ot,int}\cdot L_{ot}$$

**Compartment 5: Lymph node**

$${Vtotal}_{LN}\cdot\frac{dC_{LN}}{dt}=C_{Lungs,int}\cdot\left( L_{Lungs}-L_{PS} \right)+\frac{L_{PS}}{R_{PS}}\cdot\frac{A_{PS,int}}{{Vi}_{PS}+V_{tumor}}+C_{ot,int}\cdot L_{ot}-C_{LN}\cdot L_{total Lymph}$$

########

**Compartment 6: Pleural space**

Note: Base pleural space model (simulation profiles in Fig. 3a) is the case when no tumor is present in the pleural space.

$$\left( {Vi}_{PS}+V_{tumor} \right)\cdot\frac{dC_{PS,int}}{dt}=C_{Lungs,int}\cdot J_{PS}\cdot{Vi}_{Lungs}-\frac{L_{PS}}{R_{PS}}\cdot\frac{A_{PS,int}}{{Vi}_{PS}+V_{tumor}}-{k_{on,mac}\cdot A_{PS,int}\cdot\left( TB-\left[ CAR T and tumor cell complex \right] \right)\cdot R_{TAA}}/\left( {Vi}_{PS}+V_{tumor} \right)+k_{off,mac}\cdot[CAR T and tumor cell complex]+k_{kill}\cdot[CAR T and tumor cell complex]+k_{pro}\cdot\frac{1}{1+\frac{TB}{KI}}\cdot[CAR T and tumor cell complex]$$

# tumor growth: exponential model

# TB: total tumor burden, number of tumor cells

$$\frac{dTB}{dt}=k_{g}\cdot\left( TB-\left[ CAR T and tumor cell complex \right] \right)-k_{kill}\cdot[CAR T and tumor cell complex]$$

# tumor cell volume: 518.3 fL from 2012 Phillips et al.

# assuming (1-0.2)*100% total tumor volume is tumor cell

# 0.2 being the fraction of interstitial space of the tumor

$$V_{tumor}=\frac{518.3\times{10}^{-12}\times TB}{(1-0.2)}$$

# interactions between CAR T cells and target tumor cells: CAR T and tumor cell complex formation

$$\frac{d[CAR T and tumor cell complex]}{dt}={k_{on,mac}\cdot A_{PS,int}\cdot\left( TB-[CAR T and tumor cell complex] \right)\cdot R_{TAA}}/\left( {Vi}_{PS}+V_{tumor} \right)-k_{off,mac}\cdot[CAR T and tumor cell complex]-k_{kill}\cdot[CAR T and tumor cell complex]$$

ii) Liver tumor

**Compartment 1: Arterial blood**

$$V_{ab}\cdot\frac{dC_{ab}}{dt}=C_{Lungs,vas}\cdot Q_{Blood}-C_{ab}\cdot Q_{Liver,HA}-C_{ab}\cdot Q_{GI}-C_{ab}\cdot Q_{Spleen}-C_{ab}\cdot Q_{ot}$$

**Compartment 2: Venous blood**

$$V_{vb}\cdot\frac{dC_{vb}}{dt}=C_{ot,vas}\cdot\left( Q_{ot}-L_{ot} \right)-C_{LN}\cdot L_{total, lymph}-C_{Liver,vas}\cdot\left( Q_{Liver,HA}+Q_{GI}-L_{GI}+Q_{Spleen}-L_{Spleen}-L_{total,liver} \right)-C_{vb}\cdot\left( Q_{Blood}+L_{Lungs} \right)$$

**Compartment 3: Lungs**

# vasculature

$${Vv}_{Lungs}\cdot\frac{dC_{Lungs,vas}}{dt}=C_{vb}\cdot\left( Q_{Blood}+L_{Lungs} \right)-C_{Lungs,vas}\cdot J_{Lungs}\cdot{Vv}_{Lungs}-C_{Lungs,vas}\cdot Q_{Blood}$$

# interstitium

$${Vi}_{Lungs}\cdot\frac{dC_{Lungs,int}}{dt}=C_{Lungs,vas}\cdot J_{Lungs}\cdot{Vv}_{Lungs}-C_{Lungs,int}\cdot L_{Lungs}-k_{el}\cdot C_{Lungs,int}\cdot{Vi}_{Lungs}$$

**Compartment 4: Liver**

# vasculature

$${Vv}_{Liver}\cdot\frac{dC_{Liver,vas}}{dt}=C_{ab}\cdot\left( Q_{Liver,HA}-Q_{Tumor,HA} \right)+C_{GI,vas}\cdot\left( Q_{GI}-L_{GI}-Q_{Tumor,PV,GI} \right)+C_{Spleen,vas}\cdot\left( Q_{Spleen}-L_{Spleen}-Q_{Tumor,PV,Spleen} \right)-C_{Liver,vas}\cdot J_{Liver}\cdot{Vv}_{Liver}-C_{Liver,vas}\cdot\left( Q_{Liver,HA}-Q_{Tumor,HA}+Q_{GI}-L_{GI}-Q_{Tumor,PV,GI}+Q_{Spleen}-L_{Spleen}-Q_{Tumor,PV,Spleen}-\left( L_{total Liver}-L_{Tumor,HA}-L_{Tumor,PV} \right) \right)$$

# interstitium

$${Vi}_{Liver}\cdot\frac{dC_{Liver,int}}{dt}=C_{Liver,vas}\cdot J_{Liver}\cdot{Vv}_{Liver}-C_{Liver,int}\cdot\left( L_{total Liver}-L_{Tumor,HA}-L_{Tumor,PV} \right)/R_{Liver}$$

**Compartment 5: GI**

# vasculature

$${Vv}_{GI}\cdot\frac{dC_{GI,vas}}{dt}=C_{ab}\cdot Q_{GI}-C_{GI,vas}\cdot J_{GI}\cdot{Vv}_{GI}-C_{GI,vas}\cdot\left( Q_{GI}-L_{GI} \right)$$

# interstitium

$${Vi}_{GI}\cdot\frac{dC_{GI,int}}{dt}=C_{GI,vas}\cdot J_{GI}\cdot{Vv}_{GI}-C_{GI,int}\cdot L_{GI}$$

**Compartment 6: Spleen**

# vasculature

$${Vv}_{Spleen}\cdot\frac{dC_{Spleen,vas}}{dt}=C_{ab}\cdot Q_{Spleen}-C_{Spleen,vas}\cdot J_{Spleen}\cdot{Vv}_{Spleen}-C_{Spleen,vas}\cdot\left( Q_{Spleen}-L_{Spleen} \right)$$

# interstitium

$${Vi}_{Spleen}\cdot\frac{dC_{Spleen,int}}{dt}=C_{Spleen,vas}\cdot J_{Spleen}\cdot{Vv}_{Spleen}-C_{Spleen,int}\cdot L_{Spleen}/R_{Spleen}$$

**Compartment 7: Other tissues**

J_ot_ is derived theoretically using the following equation:

$$J_{ot}=\frac{\sum_{i} J_{i}\cdot{Vv}_{i}}{{Vv}_{ot, total}}$$

where i indicates the organs to be lumped into the other tissues compartment.

# vasculature

$${Vv}_{ot}\cdot\frac{dC_{ot,vas}}{dt}=C_{ab}\cdot Q_{ot}-C_{ot,vas}\cdot J_{ot}\cdot{Vv}_{ot}-C_{ot,vas}\cdot\left( Q_{ot}-L_{ot} \right)$$

# interstitium

$${Vi}_{ot}\cdot\frac{dC_{ot,int}}{dt}=C_{ot,vas}\cdot J_{ot}\cdot{Vv}_{ot}-C_{ot,int}\cdot L_{ot}$$

**Compartment 8: Lymph node**

$${Vtotal}_{LN}\cdot\frac{dC_{LN}}{dt}=C_{Lungs,int}\cdot L_{Lungs}+C_{GI,int}\cdot L_{GI}+C_{Spleen,int}\cdot\frac{L_{Spleen}}{R_{Spleen}}+C_{Liver,int}\cdot\frac{L_{total Liver}-L_{Tumor,HA}-L_{Tumor,PV}}{R_{Liver}}+\frac{L_{Tumor}\cdot A_{Tumor,int}}{{Vi}_{Tumor}\cdot R_{Liver}}+C_{ot,int}\cdot L_{ot}-C_{LN}\cdot L_{total Lymph}$$

########

**Compartment 9: Liver Tumor**

Note: The blood flows and lymph flow associated with the liver tumor are considered to be part of the healthy liver in the case of base liver model (simulation profiles in Fig. 3b).

# vasculature

$${Vv}_{Tumor}\cdot\frac{dC_{Tumor,vas}}{dt}=C_{ab}\cdot Q_{Tumor}-C_{Tumor,vas}\cdot J_{Tumor}\cdot{Vv}_{Tumor}-C_{Tumor,vas}\cdot\left( Q_{Tumor}-L_{Tumor} \right)$$

# interstitium

$${Vi}_{Tumor}\cdot\frac{dC_{Tumor,int}}{dt}=C_{Tumor,vas}\cdot J_{Tumor}\cdot{Vv}_{Tumor}-\frac{L_{Tumor}\cdot A_{Tumor,int}}{{Vi}_{Tumor}\cdot R_{Liver}}-{k_{on,mac}\cdot A_{Tumor,int}\cdot\left( TB-\left[ CAR T and tumor cell complex \right] \right)\cdot R_{TAA}}/{{Vi}_{Tumor}}+k_{off,mac}\cdot[CAR T and tumor cell complex]+k_{kill}\cdot[CAR T andtumor cell complex]+k_{pro}\cdot\frac{1}{1+\frac{TB}{KI}}\cdot[CAR T andtumor cell complex]$$

# tumor growth: exponential model

# TB: total tumor burden, number of tumor cells

$$\frac{dTB}{dt}=k_{g}\cdot\left( TB-\left[ CAR T and tumor cell complex \right] \right)-k_{kill}\cdot[CAR T and tumor cell complex]$$

# tumor cell volume: 518.3 fL from 2012 Phillips et al.

# assuming (1-0.2-0.15)*100% total tumor volume is tumor cell

# 0.2 being the fraction of interstitial space of the tumor; 0.15 being the fraction of the vasculature of the tumor

$$V_{tumor}=\frac{518.3\times{10}^{-12}\times TB}{(1-0.2-0.15)}$$

# interactions between CAR-T cells and target tumor cells: CAR T and tumor cell complex formation

$$\frac{d[CAR T and tumor cell complex]}{dt}={k_{on,mac}\cdot A_{Tumor,int}\cdot\left( TB-[CAR T and tumor cell complex] \right)\cdot R_{TAA}}/{{Vi}_{Tumor}}-k_{off,mac}\cdot[CAR T and tumor cell complex]-k_{kill}\cdot[CAR T andtumor cell complex]$$

**Table S1** Summary of CAR T cell therapies that have been investigated through non-systemic deliveries in mice and humans

| CAR target | Tumor host species | Cancer type | Dosing route | Results highlight | Reference |
| --- | --- | --- | --- | --- | --- |
| CEA | Mouse | Colon adenocarcinoma in peritoneal cavity | intraperitoneal (i.p.) and intravenous (i.v.) | i.p. dosing showed better efficacy than i.v., even after tumor re-challenge | (1) |
| CEA | Human | Liver metastasis | hepatic artery infusion | 1 out of 6 patients had stable disease; CAR T cells were more abundant in liver metastases than in normal liver; no grade 4 or 5 adverse events | (2) |
| CEA | Human | Liver metastasis | hepatic artery infusion | No severe CRS or neurotoxicity observed | (3) |
| Mesothelin | Mouse | Pleural tumor | i.v. and intrapleural | Earlier CAR-T proliferation with intrapleural injection at the same dose level compared to i.v.; better efficacy with intrapleural administration | (4) |
| Mesothelin | Mouse | Pleural tumor | i.v. and intrapleural | Intrapleural delivery was associated with early proliferation and efficacy while i.v. administration did not lead to expansion and TGI; TAA expression level was shown to affect CAR T trafficking to the pleural space after systemic delivery | (5) |
| Mesothelin | Human | Pleural tumor | intrapleural | No CAR T cell-related toxicities higher than grade 2 were observed; 5 with partial response and 4 with stable disease out of 19 patients | (6) |
| c-Met | Mouse | Human ovary cancer | intratumoral | Tumor stasis achieved | (7) |
| c-Met | Human | Metastatic breast cancer | intratumoral | None of the patients had CAR T-related adverse events greater than grade 1; extensive tumor necrosis; macrophage recruitment and inflammatory response within tumor | (7) |
| IL13Rα2 | Human | Glioblastoma | intracranial | No serious, unexpected adverse events encountered | (8) |
| IL13Rα2 | Human | Glioblastoma | intracranial | Well-tolerated, with manageable temporary brain inflammation; transient response observed in 2 patients; reduction in target level observed in 1 patient | (9) |
| IL13Rα2 | Human | Glioblastoma | intracavitary and intraventricular | One patient; not associated with any toxic effects of grade 3 or higher; regression of all intracranial and spinal tumors observed | (10) |
| ErbB | Mouse | Human head and neck tumor & melanoma | i.p. | Tumor growth substantially reduced | (11) |
| ErbB | Mouse | Human ovary cancer & head and neck tumor | i.v., intratumoral, and i.p. | Partial tumor regression achieved without toxicity through i.v. and intratumoral injections; with i.p. administration, tumor regressed accompanied by dose-dependent toxicity; IL-6 release can be ameliorated by depletion of macrophages | (12) |
| ErbB | Mouse | Human mesothelioma | i.p. | Tumor regression or eradication | (13) |
| ErbB | Human | head and neck squamous cell carcinoma | intratumoral | No lymphodepletion; adverse events no greater than grade 2; all 3 patients in the dose cohort of 100 million cell achieved stable disease; 1 billion cells well tolerated | (14, 15) |
| HER2 | Mouse | Human breast cancer metastatic to the brain | i.v., intratumoral, and intraventricular | 4-1BB construct led to reduced exhaustion and increased proliferation compared with CD28 construct; at least 10-fold difference in antitumor activity between intraventricular and i.v. administrations; achieved tumor eradication | (16) |
| HER2 | Mouse | Medulloblastoma | i.v. and intratumoral | Both i.v. and regional delivery resulted in eradication of xenograft tumor; i.v. delivery required 5-fold higher dose | (17) |
| TAG72 | Mouse | Peritoneal ovarian tumor | i.v. and i.p. | i.p. delivery showed significantly reduced tumor growth and extended mouse survival; efficacy further improved with repeat dosing; reduced TAG72 expression observed in recurring tumor | (18) |
| B7-H3 | Mouse | Brain tumor | i.v., intratumoral, and intraventricular | local administration of CAR T cell mediates potent antitumor effects with greater potency and safer systemic cytokine profiles, compared to i.v. administration | (19) |

**Table S2** Parameters in mPBPK-PD model for pleural tumor

| Compartments | Parameter name | Value (%CV where applicable) | Unit | Description | Source |
| --- | --- | --- | --- | --- | --- |
| Arterial blood | Q_Blood_ | 678 | mL/hr | Blood flow rate | (20) |
|  | V_ab_ | 0.8585 | mL | Volume of arterial blood | Assumed to be 50% of total blood volume in (20) |
| Venous blood | V_vb_ | 0.8585 | mL | Volume of venous blood | Assumed to be 50% of total blood volume in (20) |
| Lungs | Q_Lungs,out_ | 678 | mL/hr | Blood flow rate out of the lungs | (20) |
|  | L_Lungs_ | 0.746 | mL/hr | Lymph flow rate in the lungs from vasculature to interstitium | (20) |
|  | Vv_Lungs_ | 0.0536 | mL | Vascular volume of the lungs | (20) |
|  | Vi_Lungs_ | 0.0384 | mL | Interstitial volume of the lungs | (20) |
|  | J_Lungs_ | 1843 | 1/hr | Transmigration rate of CAR T cells in the lungs | (21) |
|  | k_eli_ | 0.84 | 1/hr | T cell elimination rate in the lungs | (22) |
| Other tissues | Q_ot_ | 678 | mL/hr | Blood flow rate into other tissues | (20) |
|  | L_ot_ | 0.904 | mL/hr | Lymph flow rate in all other tissues from vasculature to interstitium | (20) |
|  | Vv_ot_ | 1.432 | mL | Vascular volume of “other tissues” compartment | (20) |
|  | Vi_ot_ | 4.870 | mL | Interstitial volume of “other tissues” compartment | (20) |
|  | J_ot_ | 41.4 | 1/hr | Transmigration rate of CAR T cells in other tissues (derived) | (21) |
| Lymph node | L_total_Lymph_ | 1.65 | mL/hr | Total lymph flow rate in the lymph nodes | (20) |
|  | Vtotal_LN_ | 0.113 | mL | Total volume of lymph nodes | (20) |
| Tumor: pleural space | L_PS_ | 0.033 | mL/hr | Lymph flow rate in the pleural space | (23) |
|  | Vi_PS_ | 0.00728 | mL | Volume of pleural cavity | (24) |
|  | J_PS_ | 0.119 (25.6) | 1/hr | Transmigration rate in the pleural space | Estimated |
|  | R_PS_ | 2.88 (2.60) | - | Retention factor of CAR T cell in the pleural space | Estimated |
|  | k_g_ | 0.00385 (8.19) | 1/hr | Tumor exponential growth rate | Estimated |
|  | k_pro_ | 0.115 (14.9) | 1/hr | Maximum proliferation rate of CAR T cells | Estimated |
|  | KI | 3.84×10^7^ (15.4) | cells | Number of tumor cells required to inhibit 50% k_pro_ | Estimated |
|  | k_kill_ | 0.0733 (3.60) | 1/hr | Maximum tumor cell killing rate constant by CAR T | Estimated |
|  | R_TAA_ | 10^4^ | copy/cell | Copy of tumor-associated antigen per tumor cell | Assumed |
|  | k_on,mac_ | 10^6^ | M^-1^·s^-1^ | Cell-level macroscopic association rate constant | (25) |
|  | k_off,mac_ | 6.85×10^-8^ (3.74) | 1/hr | Cell-level macroscopic dissociation rate constant | Estimated |

**Table S3** Parameters in mPBPK-PD model for liver tumor

| Compartments | Parameter name | Value (%CV where applicable) | Unit | Description | Source |
| --- | --- | --- | --- | --- | --- |
| Arterial blood | Q_Blood_ | 678 | mL/hr | Blood flow rate | (20) |
|  | V_ab_ | 0.8585 | mL | Volume of arterial blood | Assumed to be 50% of total blood volume in (20) |
| Venous blood | V_vb_ | 0.8585 | mL | Volume of venous blood | Assumed to be 50% of total blood volume in (20) |
| Lungs | Q_Lungs,out_ | 678 | mL/hr | Blood flow rate out of the lungs | (20) |
|  | L_Lungs_ | 0.746 | mL/hr | Lymph flow rate in the lungs from vasculature to interstitium | (20) |
|  | Vv_Lungs_ | 0.0536 | mL | Vascular volume of the lungs | (20) |
|  | Vi_Lungs_ | 0.0384 | mL | Interstitial volume of the lungs | (20) |
|  | J_Lungs_ | 1843 | 1/hr | Transmigration rate of CAR T cells in the lungs | (21) |
|  | k_eli_ | 0.84 | 1/hr | T cell elimination rate in the lungs | (22) |
| Liver | Q_Liver,HA_ | 18.7 | mL/hr | Blood flow rate into liver through the hepatic artery | (20) |
|  | L_total,Liver_ | 0.1874 | mL/hr | Total lymph flow rate in liver from vasculature to interstitium | (20) |
|  | Vv_Liver_ | 0.298 | mL | Vascular volume of liver | (20) |
|  | Vi_Liver_ | 0.385 | mL | Interstitial volume of liver | (20) |
|  | J_Liver_ | 126.9 | 1/hr | Transmigration rate of CAR T cells in liver | (21) |
|  | R_Liver_ | 2.5 | - | Retention factor for liver | (21) |
| GI | Q_GI_ | 137 | mL/hr | Blood flow rate into GI | (20) |
|  | L_GI_ | 0.1508 | mL/hr | Lymph flow rate in GI from vasculature to interstitium | (20) |
|  | Vv_GI_ | 0.03019 | mL | Vascular volume of GI | (20) |
|  | Vi_GI_ | 0.1815 | mL | Interstitial volume of GI | (20) |
|  | J_GI_ | 18.1 | 1/hr | Transmigration rate of CAR T cells in GI | (21) |
| Spleen | Q_Spleen_ | 14.88 | mL/hr | Blood flow rate into spleen | (20) |
|  | L_Spleen_ | 0.01636 | mL/hr | Lymph flow rate in spleen from vasculature to interstitium | (20) |
|  | Vv_Spleen_ | 0.028 | mL | Vascular volume of spleen | (20) |
|  | Vi_Spleen_ | 0.0254 | mL | Interstitial volume of spleen | (20) |
|  | J_Spleen_ | 114 | 1/hr | Transmigration rate of CAR T cells in spleen | (21) |
|  | R_Spleen_ | 9.8 | - | Retention factor in spleen | (21) |
| Other tissues | Q_ot_ | 507.42 | mL/hr | Blood flow rate into other tissues | (20) |
|  | L_ot_ | 0.549 | mL/hr | Lymph flow rate in all other tissues from vasculature to interstitium | (20) |
|  | Vv_ot_ | 1.076 | mL | Vascular volume of “other tissues” compartment | (20) |
|  | Vi_ot_ | 4.278 | mL | Interstitial volume of “other tissues” compartment | (20) |
|  | J_ot_ | 16.7 | 1/hr | Transmigration rate in other tissues (derived) | (21) |
|  | J_ot_ | 94.3 (20.0) | 1/hr | Transmigration rate in other tissues | Estimated |
| Lymph node | L_total_Lymph_ | 1.65 | mL/hr | Total lymph flow rate in the lymph nodes | (20) |
|  | Vtotal_LN_ | 0.113 | mL | Total volume of lymph nodes | (20) |
| Tumor | Q_Tumor,HA_ | 2 | mL/hr | Blood flow rate into the tumor through hepatic artery | Assumed |
|  | L_Tumor,HA_ | 0.004 | mL/hr | Rate of lymph flow into tumor interstitium through hepatic artery | Assumed |
|  | Q_Tumor,PV,GI_ | 14.5 | mL/hr | Blood flow rate into the tumor through portal vein, original flow from GI | Assumed |
|  | Q_Tumor,PV,Spleen_ | 1.5 | mL/hr | Blood flow rate into the tumor through portal vein, original flow from spleen | Assumed |
|  | L_Tumor,PV_ | 0.032 | mL/hr | Rate of lymph flow into tumor interstitium through portal vein | Assumed |
|  | J_Tumor_ | 126.9 | 1/hr | Transmigration rate of CAR T cells into tumor interstitium | Assumed the same as liver |
|  | TB_0_ | 2.5×10^8^ | cells | Initial number of tumor cells | Assumed |
|  | Vv_Tumor_ | 15%  V_total Tumor_ | mL | Volume of tumor vasculature | Assumed the same proportion as liver |
|  | Vi_Tumor_ | 20%  V_total Tumor_ | mL | Volume of tumor interstitium | Assumed the same proportion as liver |
|  | k_g_ | 0.00385 | 1/hr | Tumor exponential growth rate | Pleural tumor parameter |
|  | k_pro_ | 0.115 | 1/hr | Maximum proliferation rate of CAR T cells | Pleural tumor parameter |
|  | KI | 3.84×10^7^ | cells | Number of tumor cells required to inhibit 50% k_pro_ | Pleural tumor parameter |
|  | k_kill_ | 0.0733 | 1/hr | Maximum tumor cell killing rate constant by CAR T | Assumed twice as pleural tumor |
|  | R_TAA_ | 10^4^ | copy/cell | Copy of tumor-associated antigen per tumor cell | Pleural tumor parameter |
|  | k_on,mac_ | 10^6^ | M^-1^·s^-1^ | Cell-level macroscopic association rate constant | Pleural tumor parameter |
|  | k_off,mac_ | 6.85×10^-8^ | 1/hr | Cell-level macroscopic dissociation rate constant | Pleural tumor parameter |

**Table S4** Parameters involved in fitting to pleural tumor data

| Item | Parameter name | Value | Unit | Description | Source |
| --- | --- | --- | --- | --- | --- |
| Bioluminescence readout | S_1_ | 311398 | photons/s-cell | Scaling factor for bioluminescence signal for CAR T cells | Average value of the first time points |
|  | S_2_ | 1 | photons/s-cell | Scaling factor for bioluminescence signal for tumor cells | Fixed |
|  | B_2_ | 754800 | photons/s | Baseline bioluminescence noise for tumor cells | Average value of plateaued signals |
| 1×10^6^ cells i.v. dose | TB_0_ | 5.9×10^7^ | cells | Initial number of tumor cells | Assumed |
| 0.1×10^6^ cells i.v. dose | TB_0_ | 3.0×10^8^ | cells | Initial number of tumor cells | Assumed |
| 3×10^6^ cells i.v. dose | TB_0_ | 1.0×10^8^ | cells | Initial number of tumor cells | Assumed |
| 0.1×10^6^ cells intrapleural dose | TB_0_ | 1.0×10^8^ | cells | Initial number of tumor cells | Assumed |
| 0.3×10^6^ cells intrapleural dose | TB_0_ | 6.2×10^7^ | cells | Initial number of tumor cells | Assumed |
| 1×10^6^ cells intrapleural dose | TB_0_ | 5.9×10^7^ | cells | Initial number of tumor cells | Assumed |

**References**

1. Katz SC, Point GR, Cunetta M, Thorn M, Guha P, Espat NJ, et al. Regional CAR-T cell infusions for peritoneal carcinomatosis are superior to systemic delivery. Cancer Gene Therapy. 2016;23(5):142-8. doi: <https://doi.org/10.1038/cgt.2016.14>.

2. Katz SC, Burga RA, McCormack E, Wang LJ, Mooring W, Point GR, et al. Phase I Hepatic Immunotherapy for Metastases Study of Intra-Arterial Chimeric Antigen Receptor–Modified T-cell Therapy for CEA^+^ Liver Metastases. Clinical Cancer Research. 2015;21(14):3149-59. doi: <https://doi.org/10.1158/1078-0432.Ccr-14-1421>.

3. Katz SC, Hardaway J, Prince E, Guha P, Cunetta M, Moody A, et al. HITM-SIR: phase Ib trial of intraarterial chimeric antigen receptor T-cell therapy and selective internal radiation therapy for CEA+ liver metastases. Cancer Gene Therapy. 2020;27(5):341-55. doi: <https://doi.org/10.1038/s41417-019-0104-z>.

4. Adusumilli PS, Cherkassky L, Villena-Vargas J, Colovos C, Servais E, Plotkin J, et al. Regional delivery of mesothelin-targeted CAR T cell therapy generates potent and long-lasting CD4-dependent tumor immunity. Science Translational Medicine. 2014;6(261):261ra151. doi: <https://doi.org/10.1126/scitranslmed.3010162>.

5. Skovgard MS, Hocine HR, Saini JK, Moroz M, Bellis RY, Banerjee S, et al. Imaging CAR T-Cell Kinetics in Solid Tumors: Translational Implications. Molecular Therapy - Oncolytics. 2021. doi: <https://doi.org/10.1016/j.omto.2021.06.006>.

6. Adusumilli PS, Zauderer MG, Rusch VW, Cearbhaill RE, Zhu A, Ngai DA, et al. Abstract CT036: A phase I clinical trial of malignant pleural disease treated with regionally delivered autologous mesothelin-targeted CAR T cells: Safety and efficacy. Cancer Research. 2019;79(13 Supplement):CT036. doi: <https://doi.org/10.1158/1538-7445.AM2019-CT036>.

7. Tchou J, Zhao Y, Levine BL, Zhang PJ, Davis MM, Melenhorst JJ, et al. Safety and efficacy of intratumoral injections of chimeric antigen receptor (CAR) T cells in metastatic breast cancer. Cancer Immunology Research. 2017:canimm.0189.2017. doi: <https://doi.org/10.1158/2326-6066.CIR-17-0189>.

8. Yaghoubi SS, Jensen MC, Satyamurthy N, Budhiraja S, Paik D, Czernin J, et al. Noninvasive detection of therapeutic cytolytic T cells with 18F–FHBG PET in a patient with glioma. Nature Clinical Practice Oncology. 2009;6(1):53-8. doi: <https://doi.org/10.1038/ncponc1278>.

9. Brown CE, Badie B, Barish ME, Weng L, Ostberg JR, Chang W-C, et al. Bioactivity and Safety of IL13Rα2-Redirected Chimeric Antigen Receptor CD8^+^ T Cells in Patients with Recurrent Glioblastoma. Clinical Cancer Research. 2015;21(18):4062. doi: <https://doi.org/10.1158/1078-0432.CCR-15-0428>.

10. Brown CE, Alizadeh D, Starr R, Weng L, Wagner JR, Naranjo A, et al. Regression of Glioblastoma after Chimeric Antigen Receptor T-Cell Therapy. New England Journal of Medicine. 2016;375(26):2561-9. doi: <https://doi.org/10.1056/NEJMoa1610497>.

11. Davies DM, Foster J, van der Stegen SJC, Parente-Pereira AC, Chiapero-Stanke L, Delinassios GJ, et al. Flexible Targeting of ErbB Dimers That Drive Tumorigenesis by Using Genetically Engineered T Cells. Molecular Medicine. 2012;18(4):565-76. doi: <https://doi.org/10.2119/molmed.2011.00493>.

12. van der Stegen SJC, Davies DM, Wilkie S, Foster J, Sosabowski JK, Burnet J, et al. Preclinical In Vivo Modeling of Cytokine Release Syndrome Induced by ErbB-Retargeted Human T Cells: Identifying a Window of Therapeutic Opportunity? The Journal of Immunology. 2013;191(9):4589. doi: <https://doi.org/10.4049/jimmunol.1301523>.

13. Klampatsa A, Achkova DY, Davies DM, Parente-Pereira AC, Woodman N, Rosekilly J, et al. Intracavitary ‘T4 immunotherapy’ of malignant mesothelioma using pan-ErbB re-targeted CAR T-cells. Cancer Letters. 2017;393:52-9. doi: <https://doi.org/10.1016/j.canlet.2017.02.015>.

14. Papa S, Adami A, Metoudi M, Achkova D, Schalkwyk Mv, Pereira AP, et al. Abstract CT118: T4 immunotherapy of head and neck squamous cell carcinoma using pan-ErbB targeted CAR T-cells. Cancer Research. 2017;77(13 Supplement):CT118. doi: <https://doi.org/10.1158/1538-7445.AM2017-CT118>.

15. Larcombe-Young D, Papa S, Maher J. PanErbB-targeted CAR T-cell immunotherapy of head and neck cancer. Expert Opinion on Biological Therapy. 2020:1-5. doi: <https://doi.org/10.1080/14712598.2020.1786531>.

16. Priceman SJ, Tilakawardane D, Jeang B, Murad JP, Park AK, Chang W-C, et al. Regional Delivery of Chimeric Antigen Receptor-Engineered T Cells Effectively Targets HER2+ Breast Cancer Metastasis to the Brain. Clinical Cancer Research. 2017:clincanres.2041.17. doi: <https://doi.org/10.1158/1078-0432.CCR-17-2041>.

17. Nellan A, Rota C, Majzner R, Lester-McCully CM, Griesinger AM, Mulcahy Levy JM, et al. Durable regression of Medulloblastoma after regional and intravenous delivery of anti-HER2 chimeric antigen receptor T cells. Journal for ImmunoTherapy of Cancer. 2018;6(1):30. doi: <https://doi.org/10.1186/s40425-018-0340-z>.

18. Murad JP, Kozlowska AK, Lee HJ, Ramamurthy M, Chang W-C, Yazaki P, et al. Effective Targeting of TAG72+ Peritoneal Ovarian Tumors via Regional Delivery of CAR-Engineered T Cells. Frontiers in Immunology. 2018;9(2268). doi: <https://doi.org/10.3389/fimmu.2018.02268>.

19. Theruvath J, Sotillo E, Mount CW, Graef CM, Delaidelli A, Heitzeneder S, et al. Locoregionally administered B7-H3-targeted CAR T cells for treatment of atypical teratoid/rhabdoid tumors. Nature Medicine. 2020;26(5):712-9. doi: <https://doi.org/10.1038/s41591-020-0821-8>.

20. Shah DK, Betts AM. Towards a platform PBPK model to characterize the plasma and tissue disposition of monoclonal antibodies in preclinical species and human. Journal of Pharmacokinetics and Pharmacodynamics. 2012;39(1):67-86. doi: <https://doi.org/10.1007/s10928-011-9232-2>.

21. Khot A, Satoko M, Thomas VA, Koya RC, Shah DK. Measurement and Quantitative Characterization of Whole-Body Pharmacokinetics of Exogenously Administered T Cells in Mice. Journal of Pharmacology and Experimental Therapeutics. 2019;368(3):503. doi: 10.1124/jpet.118.252858.

22. Zhu H, Melder RJ, Baxter LT, Jain RK. Physiologically Based Kinetic Model of Effector Cell Biodistribution in Mammals: Implications for Adoptive Immunotherapy. Cancer Research. 1996;56(16):3771.

23. Miserocchi G. Physiology and pathophysiology of pleural fluid turnover. European Respiratory Journal. 1997;10(1):219. doi: <https://doi.org/10.1183/09031936.97.10010219>.

24. Noppen M, De Waele M, Li R, Gucht KV, D'Haese JAN, Gerlo E, et al. Volume and Cellular Content of Normal Pleural Fluid in Humans Examined by Pleural Lavage. American Journal of Respiratory and Critical Care Medicine. 2000;162(3):1023-6. doi: <https://doi.org/10.1164/ajrccm.162.3.9910050>.

25. Faro J, Castro M, Molina-París C. A unifying mathematical framework for experimental TCR-pMHC kinetic constants. Scientific Reports. 2017;7(1):46741. doi: <https://doi.org/10.1038/srep46741>.
